# Clinical, radiological and functional outcomes in patients with SARS-CoV-2 pneumonia: a prospective observational study

**DOI:** 10.1186/s12890-021-01509-3

**Published:** 2021-04-26

**Authors:** Pietro Gianella, Elia Rigamonti, Marco Marando, Adriana Tamburello, Lorenzo Grazioli Gauthier, Gianluca Argentieri, Carla Puligheddu, Alberto Pagnamenta, Marco Pons, Tanja Fusi-Schmidhauser

**Affiliations:** 1 Department of Internal Medicine, Ospedale Regionale Di Lugano, Ente Ospedaliero Cantonale, Via Tesserete 46, 6900 Lugano, Switzerland; 2 Division of Pneumology, Ospedale Regionale Di Lugano, Ente Ospedaliero Cantonale, Lugano, Switzerland; 3 IIMSI - Radiology Department, Ospedale Regionale Di Lugano, Ente Ospedaliero Cantonale, Lugano, Switzerland; 4 Department of Intensive Care, Intensive Care Unit Ospedale Regionale Di Mendrisio, Ente Ospedaliero Cantonale, Lugano, Switzerland; 5 Unit of Biostatistics, Bellinzona, Ente Ospedaliero Cantonale, Lugano, Switzerland; 6 Division of Pneumology, University of Geneva, Geneva, Switzerland

**Keywords:** COVID-19, 3-Month outcome, Chest CT, Pulmonary function tests

## Abstract

**Background:**

All over the world, SARS-CoV-2 pneumonia is causing a significant short-term morbidity and mortality, but the medium-term impact on lung function and quality of life of affected patients are still unknown.

**Methods:**

In this prospective observational study, 39 patients with SARS-CoV-2 pneumonia were recruited from a single COVID-19 hospital in Southern Switzerland. At three months patients underwent radiological and functional follow-up through CT scan, lung function tests, and 6 min walking test. Furthermore, quality of life was assessed through self-reported questionnaires.

**Results:**

Among 39 patients with SARS-CoV-2 pneumonia, 32 (82% of all participants) presented abnormalities in CT scan and 25 (64.1%) had lung function tests impairment at three months. Moreover, 31 patients (79.5%) reported a perception of poor health due to respiratory symptoms and all 39 patients showed an overall decreased quality of life.

**Conclusions:**

Medium-term follow up at three months of patients diagnosed with SARS-CoV-2 pneumonia shows the persistence of abnormalities in CT scans, a significant functional impairment assessed by lung function tests and a decreased quality of life in affected patients. Further studies evaluating the long-term impact are warranted to guarantee an appropriate follow-up to patients recovering from SARS-CoV-2 pneumonia.

## Introduction

The severe acute respiratory syndrome-coronavirus-2 (SARS-CoV-2) infection is associated with considerable morbidity and mortality [1]. After three days, more than 75% of all infected patients have signs of viral interstitial pneumonia on chest CT scan [2]. Abnormalities in pulmonary function tests and radiological alterations were highlighted in patients affected by severe acute respiratory syndrome-coronavirus (SARS-CoV) between three to 24 months after discharge from hospital [3–10]. Since interstitial lung diseases and pulmonary vascular diseases are likely to be the most important respiratory complications, in a state-of-the-art review George PM et al. recently proposed a structured respiratory follow-up of patients with COVID-19 pneumonia [11]. However, the medium-term functional and radiological outcomes in SARS-CoV-2 survivors are still unknown.

## Aims of the study

Our study aim was to describe clinical, radiological, lung function parameters and self-reported quality of life (QoL) of patients with SARS-CoV-2 pneumonia, both at diagnosis and at three-month follow-up.

## Methods

### Case definition

Study participants were diagnosed on the result of a positive real-time reverse-transcriptase polymerase chain reaction (rRT-PCR) assay for SARS-CoV-2.

### Participants and study design

**Fig. 1:**
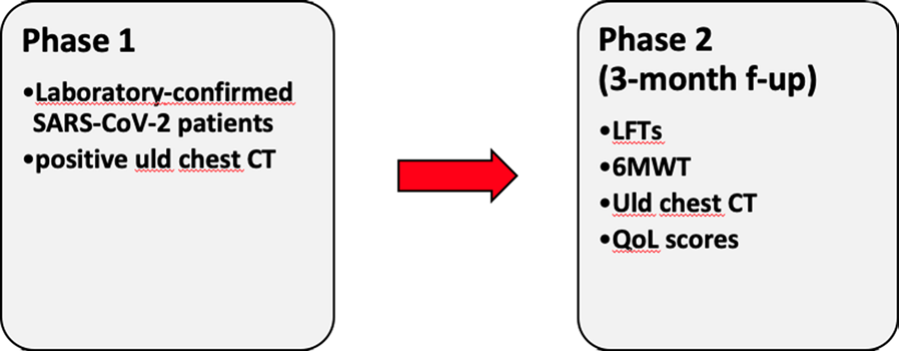
Study timeline

In this prospective observational single-center study we enrolled 39 consecutive laboratory-confirmed COVID-19 patients with pathological findings on a chest ultra-low dose (uld) CT scan performed at hospital admission between March 1 and April 15, 2020. A written informed consent was obtained from all the patients. Exclusion criteria were age < 18 years, pregnancy and absence of a written informed consent. For all included patients we collected epidemiological, clinical and laboratory data. Prior to hospital discharge a follow-up visit was planned at three months after the admission. At follow-up all patients underwent lung function tests (LFTs), 6-min Walk Test (6MWT), a chest uld CT scan and self-reported QoL questionnaires (St. George's Respiratory Questionnaire [SGRQ] and Short Form-12 [SF-12]) (Fig. 1). The study was approved by the ethics committee of Southern Switzerland and it was performed in accordance with relevant guidelines and regulations.

### Chest CT protocol

Uld CT has proven to be more sensitive for COVID-19 lesions than chest X-ray (CXR) [12] and international guidelines have also made recommendations in favour of CT for the diagnostic work-up of COVID-19 [13]. In addition, experts highlighted the issue of exposition to radiation doses and encouraged the use of low-dose CT scans [14]. All patients underwent chest uldCT in supine position at full inspiration, without intravenous contrast medium, using two multi-detector scanners: Siemens Somatom Definition Flash and Siemens Somatom Definition Edge (Siemens, Erlangen, Germany). Scan parameters for uld CT were optimized for a patient with a normal body mass index (BMI between 18.5 and 24.9 kg/m^2^) and with an effective dose varying from 0.14 to 0.5 mSv as reported in the current literature [15, 16]. Image analysis and final scores were performed by consensus by two radiologists (G.A., and C.P., with 15 and 20 years of experience in thoracic radiology, respectively) who scored independently and blinded to clinical data. Images were reviewed on a professional picture archiving and communication system (PACS) PC workstation (Philips Intellispace PACS). A semiquantitative scoring system based on the method proposed by Pan et al. [17] was used to estimate the global pulmonary involvement of all abnormalities on the basis of the area involved. For each lobe the presence of a predominant pattern for ground-glass opacity (GGO), consolidation, fibrosis or parenchymal bands was determined and each of the five lung lobes was visually scored on a scale of 0–5, with 0 indicating no involvement; 1, less than 5% involvement; 2, 5–25% involvement; 3, 26–49% involvement; 4, 50–75% involvement; and 5, more than 75% involvement. The total CT score was the sum of the individual lobar scores and ranged from 0 (no involvement) to 25 (maximum involvement). Presence of a pleural effusion, thoracic lymphadenopathy (defined as lymph node size of 10 mm in short-axis dimension) or underlying lung disease such as emphysema or fibrosis were noted but not scored.

### LFTs and QoL assessment

LFTs were conducted in the Pneumology Department using the Vyntus BODY Plethysmograph (Vyaire Medical, IL, USA) according to the European Respiratory Society (ERS) guidelines [18, 19]. We measured both static and dynamic volumes, other than performing bronchodilation tests and assessing diffusing lung capacity for carbon monoxide (DLCO). Since interstitial lung disease and pulmonary vascular diseases are considered the most important lung complications of COVID-19 [11], we defined as abnormal LFT the presence of a DLCO < 75% than predicted and/or of a TLC < 80% than predicted. Thereafter, patients underwent a 6MWT and self-reported QoL questionnaires (SGRQ and SF-12) were submitted [20, 21]. While the SGRQ is widely used to evaluate QoL in patients with respiratory diseases, SF-12 provides a more global assessment of patients, especially with regard to their role limitations as a result of emotional problems, mental health, bodily pain, and general health perception.

### Statistical analysis

Quantitative data were summarised as median with interquartile range (IQR) or mean with standard deviation (SD), whereas qualitative data as absolute numbers with percentages. Comparisons between groups (patients with radiological improvement versus patients without radiological improvement on the basis of the total CT score) were performed with the Kruskal–Wallis test, chi-squared test or Fisher exact test, as appropriate. All tests were performed two-sided and a *p* value < 0.05 was considered statistically significant. Statistical analysis was performed using Stata version 15 (StataCorp LP, College Station, TX, USA).

## Results

An overview of participants’ main demographic and clinical characteristics is shown in Table 1. On admission, all enrolled patients presented abnormalities on CT scans. The most frequently reported abnormal findings were GGO (89.5% of all participants), followed by fibrous bands (71.8%) and consolidations (43.6%). According to the CT score, the mean lobe injury was 2.45 and the overall lung injury was 12.26. At three months, 82% of the cohort had persisting abnormalities on CT scans, mostly fibrous bands (69.2%) and GGO (58%), the mean lobe injury was 1.39 and the overall lung injury was 6.95 (Tables 2, 3). At follow-up, we reported a statistically significant reduction in the CT score, both overall and per lobe and in GGO and consolidation incidence, while fibrous bands remained almost unaltered. LFTs abnormalities (i.e. reduced DLCO and/or restriction) were found in 25 (64.1%) patients, specifically a reduced DLCO (< 75% than predicted) in 22 (56.4%) patients and restriction in 3 (7.7%) patients. Furthermore, an overall homogeneous low effort SpO_2_ during 6MWT was also noted (91.3% ± 3.5) (Table 4).

**Table 1:** Clinical characteristics on admission

Parameters	Normal range	Over all (39)	CT improving at 3 month (31)	CT not improving at 3 month (8)
Age (years, median and IQR)	≥ 18	62.5 (51.3–71)	59.2 (50.2–71)	69.4 (60.2–71.7)
Sex (female, n and %)		9 (23.1)	7 (22.6)	2 (25)
BMI > 25 kg/m^2^ (n and %)		27 (69.2)	21 (67.7)	6 (75)
Active smokers (n and %)		3 (7.7)	2 (6.4)	1 (12.5)
Previous smokers (n and %)		12 (30.8)	8 (25.8)	4 (50)
Smoking burden (p/y, mean ± SD)		10.4 ± 16.6	7.8 ± 14.9	20.7 ± 19.9
Allergies (n and %)		11 (28.2)	9 (29)	2 (33.3)
Flu vaccination on adm. (n and %)		12 (30.8)	9 (29)	4 (50)
Pneumococcal vaccination on adm. (n and %)		1 (2.6)	0 (0)	1 (12.5)
Length of stay (days, median and IQR)		15 (12–22)	15 (11–21)	15.5 (12–28.7)
Hypertension (n and %)		11 (28.2)	9 (29)	5 (62.5)
Diabetes (n and %)		5 (12.8)	4 (12.9)	1 (12.5)
Cardiovascular diseases (n and %)		7 (17.9)	5 (16.1)	2 (33.3)
Coronary heart disease (n and %)		4 (10.2)	2 (6.4)	2 (33.3)
Chronic respiratory diseases (n and %)		8 (20.5)	6 (19.4)	2 (33.3)
COPD (n and %)		3 (7.7)	2 (6.4)	1 (12.5)
Asthma (n and %)		5 (12.8)	4 (12.9)	1 (12.5)
Chronic kidney disease (n and %)		3 (7.7)	2 (6.4)	1 (12.5)
Malignancy (n and %)		4 (10.2)	3 (9.7)	1 (12.5)
Intensive care unit admission (n and %)		10 (25.6)	9 (29)	1 (12.5)
Invasive mechanical ventilation (n and %)		7 (17.9)	6 (19.4)	1 (12.5)
Rehab. after discharge (n and %)		7 (17.9)	6 (19.4)	1 (12.5)
Peak PCR (mg/l) (mean ± SD)	1–5	185.7 ± 147.4	178.5 ± 137.4	213.4 ± 189.6
Peak LDH (U/l) (mean ± SD)	< 500	653.2 ± 348.5	693.4 ± 364.8	502.4 ± 240.3
Peak leukocytes (G/l) (mean ± SD)	4.2–10	8.9 ± 4.8	8.3 ± 3.8	11.1 ± 7.5
Peak lymphopenia (G/l) (mean ± SD)	1.5 – 2.5	0.7 ± 0.2	0.7 ± 0.2	0.6 ± 0.3
Peak thrombopenia (G/l) (mean ± SD)	150–400	185.5 ± 81.9	187.6 ± 72.6	177 ± 117
Peak d-dimer (mg/l) (mean ± SD)	< 0.5	4.8 ± 10	5.3 ± 11.5	2.9 ± 2.9
Lympho. on adm. (G/l) (mean ± SD)	1.5–2.5	0.8 ± 0.3	0.9 ± 0.3	0.67 ± 0.3
Leuko. on adm. (G/l) (mean ± SD)	4.2–10	5.5 ± 2.3	5.5 ± 2.3	5.5 ± 2.4
Thrombo on adm. (G/l) (mean ± SD)	150–400	189.9 ± 74.7	190 ± 64.2	189.4 ± 112.4
PaO2 on adm. (kPa) (mean ± SD)	> 8	9.3 ± 1.4	9.3 ± 1.5	9.2 ± 0.6
nt-proBNP on adm. (ng/l) (mean ± SD)	< 450	275.7 ± 253.7	229.5 ± 219.3	488 ± 318.4
D-dimer (mg/l) (mean ± SD)	< 0.5	1.1 ± 0.8	1.2 ± 0.9	0.9 ± 0.6
Antibiotics (n and %)		24 (61.5)	21 (67.7)	8 (37.5)
Hydroxychloroquine (n and %)		32 (82)	24 (77.4)	8 (100)
Remdesevir (n and %)		2 (5.1)	2 (6.4)	0 (0)
Tocilizumab (n and %)		4 (10.2)	4 (12.9)	0 (0)
Lopinavir-Ritonavir (n and %)		21 (53.8)	19 (61.3)	2 (25)
ACE-I, ARB treatment (n and %)		11 (28.2)	8 (25.8)	3 (37.5)
Anticoag. on adm. (n and %)		4 (10.2)	2 (6.4)	2 (25)
Antiplt. on adm. (n and %)		7 (17.9)	6 (19.4)	1 (12.5)
GGO on adm (n and %)		34 (89.5)	27 (87.1)	7 (87.5)
Consolidations on adm (n and %)		17 (43.6)	15 (48.4)	2 (25)
Fibrous bands on adm (n and %)		28 (71.8)	22 (71)	6 (75)

**Table 2:** Radiological characteristics on admission and at three months

Parameters	CT on admission (39)	CT at 3 months (39)	*P* value
GGO (n and %)	34 (89.5)	23 (58)	0.006
Consolidations (n and %)	17 (43.6)	1 (2.6)	< 0.0001
Fibrous bands (n and %)	28 (71.8)	27 (69.2)	0.81
Pathological CT scans (n and %)	39 (100)	32 (82)	0.01

**Table 3:** CT score (0–5) per lobe and overall (0–25) on admission and at three months

Parameters	CT on admission	CT at 3 months	*P* value
Right upper lobe (mean ± SD)	2.5 ± 1.2	1.4 ± 1.2	< 0.0001
Middle lobe (mean ± SD)	2.0 ± 1.3	1.2 ± 1.1	0.0002
Right lower lobe (mean ± SD)	2.7 ± 1.1	1.2 ± 1.2	< 0.0001
Left upper lobe (mean ± SD)	2.4 ± 1.4	1.4 ± 1.3	< 0.0001
Left lower lobe (mean ± SD)	2.7 ± 1.0	1.5 ± 1.3	< 0.0001
CT score per lung lobe (mean ± SD)	2.4 ± 1.2	1.4 ± 1.2	< 0.0001
CT score overall (mean ± SD)	12.3 ± 4.6	6.9 ± 5.0	< 0.0001

**Table 4:** LFTs results and clinical evaluation at three months

Parameters	Normal range	Overall (39)	CT improving at 3 month (31)	CT not improving at 3 month (8)	*P* value
FEV 1 (l) (mean ± SD)		2.9 ± 0.7	3.0 ± 0.7	2.6 ± 0.7	0.045
FEV 1 (% ± SD)		93.4 ± 16.1	95.1 ± 14.8	89.6 ± 15.6	0.52
FVC (l) (mean ± SD)		3.7 ± 0.9	3.8 ± 0.9	3.5 ± 1.1	0.97
Obstruction (n and %)		3 (7.7)	1 (3.2)	2 (25)	0.10
Restriction (n and %)		3 (7.7)	2 (6.5)	1 (12.5)	0.50
Abnormal DLCO (n and %)		22 (56.4)	18 (58.1)	4 (50)	0.71
DLCO (%, mean ± SD)	> 75	71.3 ± 15.5	70.5 ± 11.5	74.1 ± 26.5	0.62
LFTs abnormalities (n and %)		25 (64.1)	20 (64.6)	5 (62.5)	1
6MWT (m, mean ± SD)		539.3 ± 102.8	545.8 ± 96.6	514 ± 134.1	0.33
SpO_2_ at rest at 3 month (%, mean ± SD)	95–100	95.6 ± 1.6	95.7 ± 1.7	95 ± 1.2	0.10
SpO_2_ effort at 3 month (%, mean ± SD)	95–100	91.3 ± 3.5	91.2 ± 3.9	91.4 ± 1.9	0.43
mMRC score (≥ 2) at 3 month (n and %)		6 (15.4)	4 (12.9)	2 (25)	0.58

Concerning patients-reported QoL, 31 patients (79.5%) presented an abnormal total score on the St. George’s Respiratory Questionnaire and all patients reported an abnormal SF-12 score. The mean St. George total score was 16.97 (normal value 6) and the mean SF-12 score was 30.97 (normal value 50). These results show a significantly altered QoL, comparing to the general population (*p* < 0.0001) (Table 5). A sub-analysis of the SGRQ highlights the socio-economic impact of COVID-19: in effect, at 3-month follow-up, 4 patients (10.2%) declared to have stopped their working activity due to the effects of COVID-19. On the other hand, 32 (82%) of patients continued to work without complaining any reduction of their performance. The remaining 3 patients (7.8%) declared that they did not work at all both before and after COVID-19 – 2 patients due to invalidity and 1 patient declared herself a housewife.

**Table 5:** QoL assessment at three months

Parameters	Healthy subjects	Overall (39)	*P* value	CT improving at 3 month (31)	CT not improving at 3 month (8)	*P* value
St George symptoms (mean ± SD)	12	21.7 ± 18.6	0.0015	2 ± 19.4	20.4 ± 16	0.83
St George activity (mean ± SD)	9	27.1 ± 24.1	< 0.0001	23.2 ± 21.4	42.1 ± 29.5	0.09
St George impact (mean ± SD)	2	9.8 ± 17.9	0.8367	7.2 ± 15.8	20.0 ± 22.7	0.13
St George total (mean ± SD)	6	17 ± 17.4	< 0.0001	14.44 ± 15.3	26.8 ± 22.4	0.15
Abnormal St. George total (n and %)		31 (79.5)	NA	24 (77.4)	7 (87.5)	1
SF-12 score (mean ± SD)	50	31 ± 1.6	< 0.0001	30.9 ± 1.7	31.3 ± 1.2	0.60
Abnormal SF-12 score (n and %)		39 (100)	NA	31 (100)	8 (100)	1

In the univariate analysis we did not find any variable as predictor of favorable CT improvement.

Regarding the clinical significance of the CT scan improvement, we have found a positive association between FEV1 volume and CT scan improvement, with a difference of up to 20% in FEV1 volume between the two subgroups. Finally, patients with CT scan improvement did not report statistically significant better scores in QoL questionnaires.

## Discussion

In our cohort of patients recovering from SARS-CoV-2 pneumonia, 82% of patients still present radiological abnormalities (mostly fibrous bands and GGO) and 64.1% show impairment in LFTs, mostly a reduced DLCO at a three-month follow-up. In addition, 79.5% of all patients report an abnormal score on the St. George’s Respiratory Questionnaire and all patients have an abnormal SF-12 score. These results reveal the extent of the noxious effects of SARS-CoV-2 pneumonia on survivors.

Other authors have recently reported mid-term sequelae in patients with SARS-CoV-2 pneumonia, specifically Tabatabae et al. report residual disease in CT scans in 42.3% of patients at 3 months, mostly in the subgroup admitted to an intensive care unit (ICU) [22], Daher et al. report persistent fatigue without any abnormality in lung function at 6 weeks in a cohort of patients who did not require mechanical ventilation [23].

We observed that while GGO are consistently reduced and consolidations tend to resolve after three months, fibrous lesions remain almost unchanged, a find that might be the expression of a pre-existing lung injury. In literature, GGO and consolidations are reported to increase in the first two to three weeks after admission [24] and the development of lung fibrosis was described as early as at one-week [25] and at one-month [26], regardless of the severity of COVID-19. Nevertheless, the fibrotic burden in our cohort at baseline was very impressive, being as high as 71.8%.

Lung functions abnormalities in SARS-CoV-2 survivors have recently been reported [27, 28], mostly in convalescent patients after COVID-19 pneumonia. The most frequently identified abnormalities were restriction and reduced DLCO. In our study the most frequent functional abnormality was reduced DLCO (< 75% of predicted), found in 22 (56.4%) patients. The mean DLCO value was 71.3% ± 15.5 of the predicted values. Moreover, pulmonary restriction was noted in 3 (7.7%) patients. The univariate analysis showed a significant decrease in FEV1 volume in the subgroup of patients without radiological improvement, with volume reduction of up to 20%. Whether this is associated with a future development of a restrictive or obstructive pattern it is actually unknown. In heavy smokers it has been described that FEV1 decline is a marker of chronic obstructive pulmonary disease (COPD) development [29], but further research on the role of FEV1 decline meaning in predisposing to airway or lung diseases is indeed warranted. Nonetheless, we could not identify a significance between overall LFTs abnormalities and CT scan improvement, as the events per covariate were too few to draw a final conclusion.

The exploratory analysis did not show any predictor of CT scan improvement on the basis of CT score. Many variables have shown a promising trend toward significance in forecasting CT scan improvement: younger age, female sex, fewer overall burden of smoking, absence of hypertension, higher lymphocyte count at admission, lower N-terminal pro-brain natriuretic peptide (nt-proBNP) at admission, therapy with lopinavir/ritonavir. Similar associations were reported in other studies [30]. Moreover, patients with radiological improvements tend to have less airways obstruction, a higher SpO_2_ at rest and a better perceived quality of life, as assessed by lower total scores on the St. George Respiratory Questionnaire. Nevertheless, p-values did not reach significance for any of these aforementioned variables probably as a consequence of the small sample size.

As reported for SARS infection [31, 32] and influenza [33, 34], it seems that the SARS-CoV-2 infection provokes long-term consequences. In our analysis we report the findings in our cohort of patients with SARS-CoV-2 pneumonia up to three months after the hospital admission: a longer follow-up could be of use to clarify the long-term effects of COVID-19 on lung function and perceived quality of life.

Our study has several limitations. The study is monocentric, the sample is relatively small, and the three-month follow-up could be considered not sufficient to fully elucidate the long-term consequences. Furthermore, every patient in the study cohort presented with pneumonia at diagnosis and approximately 75% of the included patients were not admitted to ICU, thus the external validity of our results is limited for asymptomatic or critically ill patients. Nonetheless, we believe that this study adds some important information about the medium-term outcomes of SARS-CoV-2 pneumonia, while justifying further research focusing on long-term consequences of this condition.

## Conclusions

Three months after recovering from SARS-CoV-2 pneumonia, significant radiological abnormalities and LFTs impairment were found respectively in about 80% and 64% of patients. Moreover, about 80% of patients reported a poor perceived health due to respiratory symptoms and every patient presented an overall decreased quality of life.

According to these results, considering the relevant impairment in survivors and the great number of people recovering from SARS-CoV-2 pneumonia all over the world, a longer follow-up is warranted to assess and clarify the long-term consequences of this condition.

## Data Availability

The datasets used and analyzed during the current study are available from the corresponding author on reasonable request.
